# Comparison of Percutaneous Internal Ring Suturing (PIRS) versus Open Ligation of the Patent Processus Vaginalis for the Treatment of Communicating Pediatric Hydrocele

**DOI:** 10.3390/children11040437

**Published:** 2024-04-05

**Authors:** Zenon Pogorelić, Petar Stanić, Marko Bašković

**Affiliations:** 1 Department of Pediatric Surgery, University Hospital of Split, Spinčićeva Ulica 1, 21000 Split, Croatia; 2 Department of Surgery, School of Medicine, University of Split, Šoltanska Ulica 2a, 21000 Split, Croatia; 3 Department of Pediatric Surgery, Children’s Hospital Zagreb, Ulica Vjekoslava Klaića 16, 10000 Zagreb, Croatia; 4 Scientific Centre of Excellence for Reproductive and Regenerative Medicine, School of Medicine, University of Zagreb, Šalata 3, 10000 Zagreb, Croatia

**Keywords:** percutaneous internal ring suturing, PIRS, hydrocele, communicating hydrocele, laparoscopy, minimally invasive surgery, hernia, children

## Abstract

Background: Although the laparoscopic approach become standard for the treatment of many surgical conditions many studies still debating whether laparoscopic surgery has significant advantages over open surgery in regards to hernia or hydrocele treatment. This study aimed to evaluate the outcomes of treatment of treatment of communicating hydrocele in pediatric patients between laparoscopic percutaneous internal ring suturing (PIRS) and open ligation of the patent processus vaginalis (PPV). Methods: The medical records of pediatric patients who underwent surgery for communicating hydrocele between 1 January 2019 and 1 January 2024 were retrospectively reviewed. The primary objective of the study is to investigate the outcomes of treatment (complications and recurrence rates) of communicating hydrocele in children between laparoscopic and open surgical approaches. Secondary outcomes of the study are the duration of surgery and anesthesia, length of hospital stay (LOS), frequency of hospital readmissions (ReAd) and unplanned return to the operating room (uROR). Results: A total of 198 children underwent surgery for a communicating hydrocele (205 repairs, as 7 cases were bilateral) and were included in the study. Of these, 87 children underwent a PIRS, while the remaining 111 cases underwent open ligation of the PPV. No recurrence of the hydrocele was observed in any of the study groups. Intraoperative complication (epigastric vein injury) was noted in two cases in both groups (2.3% vs. 1.8%, *p* > 0.999). A slightly higher number of postoperative complications was observed in the open group (*n* = 7, 6.3%) compared to the PIRS group (*n* = 2, 2.3%) (*p* = 0.190). The median duration of surgery (15 min (IQR 10, 17) vs. 21 min (IQR 15, 25); *p* < 0.001) and anesthesia (30 min (IQR 25, 40) vs. 40 min (IQR 35, 40); *p* < 0.001) were significantly lower in the PIRS group compared to open ligation of the PPV. In addition, a significantly shorter median of LOS was observed in the PIRS group compared to the open PPV group (9 h (IQR 8, 12) vs. 24 h (IQR 12, 24; *p* < 0.001). No cases of ReAd and uROR were observed in any of the study groups. Conclusions: PIRS is a safe and effective laparoscopic technique that can be used in the treatment of communicating hydrocele in children. PIRS showed excellent outcomes and a low incidence of complications and recurrences, comparable to traditional open surgery.

## 1. Introduction

Ambroise Paré first described hydrocele testis in the 15th century. It is defined as an abnormal collection of serous fluid in the area between the parietal and visceral layers of tunica vaginalis [1,2]. It may be congenital or acquired (primary/idiopathic and secondary) [3,4]. Congenital patent processus vaginalis, which permits fluid flow between the peritoneal and tunica cavities, is the primary cause of hydrocele in children. The frequency is about 5.7% [5]. These hydroceles often disappear spontaneously in 18 to 24 months [6]. Thus, unless a hernia cannot be excluded, most surgeons may avoid a hydrocele operation within the first two years of life [7].

Traditional open repair entails performing an inguinal incision, dissecting the inguinal canal, high ligation of the patent processus vaginalis (PPV), and draining the fluid or window created in the tunica vaginalis. However, laparoscopic closure of the internal orifice of the PPV became an option for the treatment of hydroceles in children [7,8,9,10]. Less damage to the spermatic cord and spermatic duct, more aesthetically pleasing incisions, and the ability to identify and treat contralateral PPV (cPPV) and other anomalies are some of the benefits of laparoscopic surgery over open surgery [9,10,11]. This technique feels easier than laparoscopic hernia repairs because, unlike children with inguinal hernias, the peritoneum is usually not thickened and is easier to dissect from the spermatic elements [8]. Most of the previous studies favor single-port single-incision laparoscopic treatment of hernias and hydroceles [5,11,12,13]. Although for certain conditions the laparoscopic approach still cannot completely replace traditional open surgery, advocates of laparoscopic techniques believe that, over time, open approaches will become fewer and fewer [12,14,15]. We are still witnessing studies debating whether laparoscopic surgery has significant advantages over open surgery [14,15,16,17,18,19,20].

Since Dariusz Patkowski introduced percutaneous internal ring suturing (PIRS) almost 20 years ago, the technique is gaining more and more popularity among pediatric surgeons [21]. In PIRS, only a single umbilical port is used for the introduction of a laparoscope. This technique involves the percutaneous closure of the internal inguinal ring using a spinal needle under the control of the laparoscope, which provides better visualization of the abdominal cavity and the possibility of detecting other abnormalities or repairing the hydrocele on the contralateral side [17,22,23,24].

This single-center, retrospective five-year study aimed to evaluate the success of surgical treatment of communicating hydrocele in children between laparoscopic PIRS and open surgical approaches.

## 2. Materials and Methods

### 2.1. Patients

**Figure 1:**
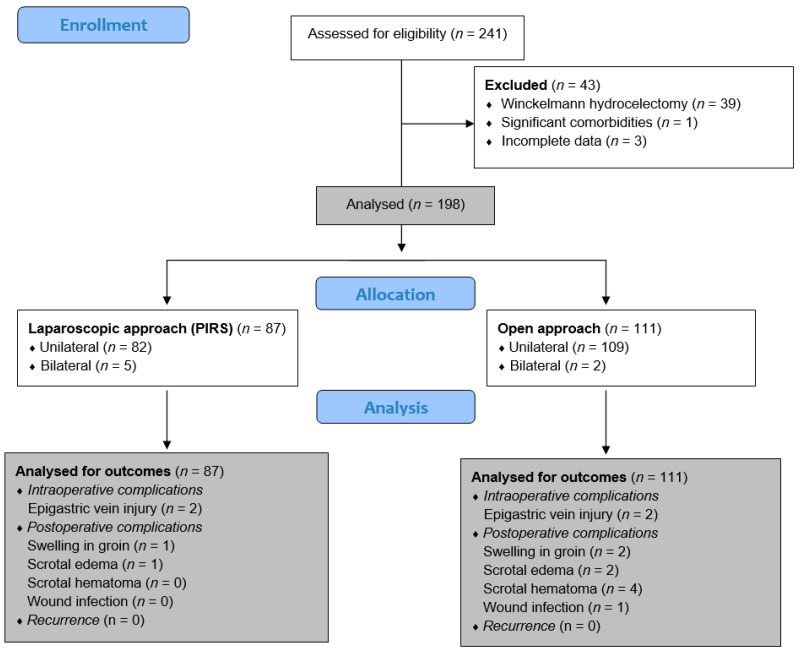
Flow chart of the study.

The medical records of pediatric patients who underwent surgery for communicating hydrocele in the Department of Pediatric Surgery at the University Hospital of Split between 1 January 2019 and 1 January 2024 were retrospectively reviewed. A total of 241 children who underwent surgery for hydrocele were identified, but 43 of them were excluded from further analysis because they met one or more exclusion criteria. Finally, 198 children (205 hydroceles) met the inclusion criteria and were included in the study. The inclusion criteria were pediatric patients older than 2 years of age who underwent open transinguinal ligation of the PPV or laparoscopic percutaneous internal ring suture (PIRS) for unilateral or bilateral hydrocele. Patients older than 17 years, patients who had undergone transscrotal Winkelmann hydrocelectomy procedure, patients with a follow-up period of fewer than three months or patients with incomplete data in the medical records were excluded from the study. The flow chart of this study is shown in Figure 1.

### 2.2. Ethical Aspects

This study was conducted in accordance with the 1964 Helsinki Declaration of the World Medical Association and its subsequent amendments or comparable ethical standards. The Institutional Review Board of the University Hospital of Split has approved the study (approval number: 500-03/23-01/220; date of approval: 27 November 2023).

### 2.3. Outcomes of the Study

The primary objective of this study is to investigate the factors of treatment outcome (complications and recurrence rates) of communicating hydrocele in children between laparoscopic and open surgical approaches. The Clavien Dindo classification was used to rank the severity of surgical complications [25]. Secondary outcomes of the study are the duration of surgery and anesthesia, length of hospital stay (LOS), frequency of hospital readmissions and postoperative pain rate. The frequency of unplanned return to the operating room (uROR) [26] and the number of readmissions within 30 days after the index operation (ReAd) [27] were examined and used as indicators of the quality of care.

### 2.4. Study Design

According to the literature and the guidelines of our department, surgery is recommended if the hydrocele persists after the second year of life [28,29]. Patients were divided into two study groups according to the surgical approach used. The first study group (*n* = 87) consisted of the patients who were treated laparoscopically and received a percutaneous internal ring suture technique, while the patients in the second group (*n* = 111) were treated with an open approach. The choice of surgical technique was based on the preferences of the parents and the surgeon. A total of six pediatric surgeons were involved in each of the two procedures. All surgeons involved in this study were allowed to perform both the open and laparoscopic approaches. The following demographic and clinical data were collected for each patient enrolled in the study: age, weight, height, body mass index (BMI), lateralization of the hydrocele, concomitant diseases and the American Society of Anesthesiologists (ASA) classification. The hydroceles in the laparoscopic group were classified with the laparoscope on the hydrocele side according to the Chang classification [30]. For each patient, the duration of surgery and anesthesia, ReAd and uROR rates and length of hospital stay were recorded. In addition, the recurrence rates and intraoperative (blood vessel injury) and postoperative (groin swelling, scrotal edema, scrotal hematoma and wound infection) complications were recorded.

### 2.5. Surgical Techniques

All patients were operated on under general anesthesia, with a laryngeal mask [31]. During the procedure, all patients were placed in the supine position with both arms crossed at the side.

#### 2.5.1. Percutaneous Internal Ring Suturing (PIRS)

**Figure 2:**
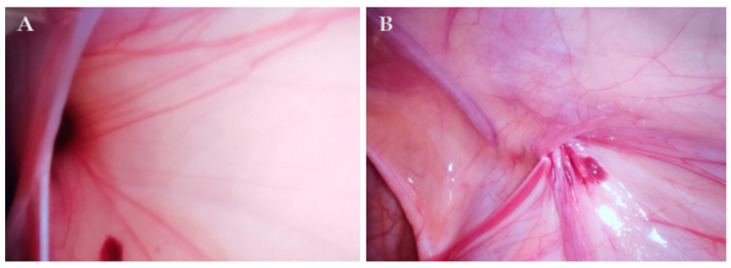
Hydrocele type II A (wide open ring with direct communication to peritoneal cavity) in a 3-year-old patient: (**A**) before surgery; (**B**) after surgery.

**Figure 3:**
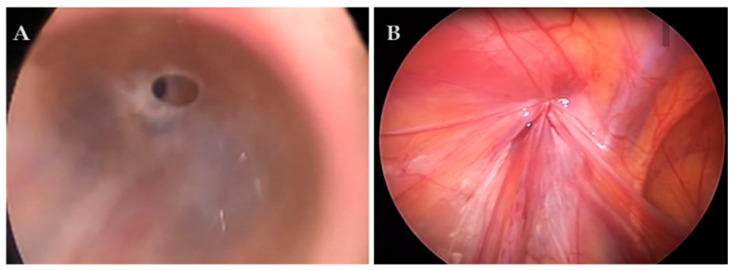
Hydrocele type II C (pinhole with direct communication to peritoneal cavity) in a 5-year-old patient: (**A**) before surgery; (**B**) after surgery.

In cases where there was a wide communication between the PPV and the abdominal cavity (hydrocele type IIA; Figure 2) or a so-called pinhole (hydrocele type IIC; Figure 3), fluid was drained from the scrotum into the abdominal cavity and the standard PIRS procedure described in our previous publication was performed [32]. In cases where the opening was covered by a peritoneal seal (hydrocele type IIB) or in a hydrocele type III, an additional 3.5 m trocar was inserted on the lateral abdominal wall on the opposite side to the hydrocele, and dissection of the peritoneum was performed followed by hydrocelectomy and a standard PIRS procedure. In the case of a bilateral hydrocele, the same procedure was performed on the other side.

#### 2.5.2. Ligation of Patent Processus Vaginalis (PPV)

Using the inguinal approach, the aponeurosis of the external abdominal oblique muscle is incised horizontally 1–2 cm above the external inguinal ring over a length of 2–3 cm along the cord to the inner ring. The cord structures are dissected away from the PPV. The transverse fascia remains intact. The PPV is then highly ligated with absorbable suture (Vycril™ 4-0, polyglactin 910, Ethicon^®^, Cincinnati, OH, USA). After ligation, the PPV is resected above the suture and the fluid is drained from the scrotum. At the end of the procedure, the wound is closed in anatomical layers. In the case of a bilateral hydrocele, the same procedure was performed on the other side.

### 2.6. Postoperative Protocol and Follow-Up

Postoperative care was standardized and the same in both groups. Most patients were started on oral nutrition within two hours of surgery. For pain relief, paracetamol (Perfalgan, Bristol-Myers Squibb S.r.l., Agen, France) was administered at a dose of 10–15 mg/kg or ibuprofen (Brufen, Mylan, Zagreb, Croatia) at a dose of 10 mg/kg. Patients were discharged from the hospital when they were fever-free, pain-free and tolerated oral nutrition well. The patients were followed further in our outpatient clinic. The skin sutures or skin adhesive tapes were removed 5 to 7 days after the procedure. The follow-up program included physical examinations 1, 6 and 12 months after the procedure and once a year to determine the presence of recurrence or complications.

### 2.7. Statistical Analysis

Statistical Package for the Social Sciences software version 28.0 (IBM Corp, Armonk, NY, USA) and Microsoft Excel for Windows version 11.0 (Microsoft Corporation, Redmond, WA, USA) were used for the statistical analysis. The distribution of quantitative data was expressed by median and interquartile range (IQR), while absolute numbers and percentages were used to describe categorical data. A nonparametric Mann–Whitney U test was used to compare continuous variables, while the chi-square test was used to compare categorical variables. A two-tailed Fisher’s exact test was used when the frequency of events in a given cell was low. All *p*-values less than 0.05 were considered significant.

## 3. Results

### 3.1. Demographic Characteristics and Clinical Data of the Patients

A total of 198 children underwent surgery for a communicating hydrocele (205 repairs, as 7 cases were bilateral) and were included in the study. Of these, 87 children underwent a laparoscopic procedure (PIRS), while the remaining 111 cases underwent open ligation of the PPV. The patients in the PIRS group were slightly younger (median 3 years (IQR 3, 4)) compared to the patients who underwent open PPV (median 4 years (IQR 3, 5)) (*p* = 0.002), but this has no clinical significance. In addition, they had slightly lower values for body weight (*p* < 0.001) and height (*p* < 0.001) compared to the open group. There were no statistically significant differences between the groups in terms of BMI (*p* = 0.447), lateralization of the hydrocele (*p* = 0.174), ASA classification (*p* = 0.891) and comorbidities (*p* = 0.771). The demographic characteristics and clinical data of the patients from both groups are shown in Table 1.

The hydroceles in the laparoscopic group were classified according to the shape of the internal inguinal ring on the hydrocele side. No case of hydrocele type I was found in this group. Type II hydrocele was found in the majority of cases (94.6%), while type III hydrocele was found in the remaining 5.4% of cases (Table 2).

### 3.2. Outcomes of Treatment of the Patients

The outcomes of the treatment were compared between the study groups. With regard to the primary outcome of the study, no recurrence of the hydrocele was observed in any of the study groups. Intraoperative complication (epigastric vein injury) was noted in 2 cases in both groups (2.3% vs. 1.8%, *p* > 0.999). Although there was a higher number of postoperative complications in the open group (*n* = 7, 6.3%) compared to the PIRS group (*n* = 2, 2.3%), there were no statistically significant differences (*p* = 0.190). All postoperative complications were classified as Clavien–Dindo grade I and treated conservatively. Local application of cold was performed for hematomas, edema or swelling in the groin. In the case of wound infections, the wound was opened and drained.

With regard to secondary outcomes, the median duration of surgery (15 min (IQR 10, 17) vs. 21 min (IQR 15, 25); *p* < 0.001) and anesthesia (30 min (IQR 25, 40) vs. 40 min (IQR 35, 40); *p* < 0.001) were significantly lower in the PIRS group compared to open ligation of the PPV. In addition, a significantly shorter median of LOS was observed in the PIRS group compared to the open PPV group (9 h (IQR 8, 12) vs. 24 h (IQR 12, 24); *p* < 0.001). No cases of ReAd and uROR were observed in any of the study groups. Outcomes of the treatment of patients operated on for a hydrocele are shown in Table 3.

## 4. Discussion

In this study, treatment outcomes, duration of surgical procedure and anesthesia, length of hospital stay, frequency of hospital readmissions, and postoperative pain rate were evaluated between laparoscopic (PIRS) and open surgical approaches in the treatment of communicating hydrocele in children. The results showed no superiority between the two study groups in terms of postoperative complications. There were also no cases of ReAd and uROR in either study group. However, a significantly shorter duration of surgery and anesthesia, as well as a shorter median length of stay, were observed in the PIRS group compared to the open PPV group.

Laparoscopic surgery has developed rapidly in the field of pediatric surgery as it is minimally invasive to the abdominal cavity of children. Several techniques for laparoscopic repair of inguinal hernias have been introduced and laparoscopic hernia repair is now commonly performed. However, there are few studies on laparoscopic hydrocele in children [33,34]. In regards to pediatric hydrocele treatment, surgeons have stated that laparoscopic surgery is only indicated for the treatment of communicating hydrocele. The surgical principle of laparoscopic treatment of communicating hydrocele in children is the anatomic closure of the PPV, which is consistent with the treatment of inguinal hernias in children. There are two categories of laparoscopic suturing techniques corresponding to the suturing techniques of the internal inguinal ring: extracorporeal or intracorporeal sutures and knot techniques. Percutaneous internal ring suturing is one of the extracorporeal suturing techniques. As there is no intracorporeal suture, this surgical technique is technically simpler and has a shorter learning curve [35,36,37]. Several advantages have been reported, including shorter operative time, better cosmetic results, no need for tracheal intubation, lower recurrence rates, and a lower likelihood of complications [22,35,38]. The PIRS technique has been reported to cause significantly lower levels of pain and inflammatory stress responses [17].

In this study, the hydroceles in the PIRS group were classified according to Chang’s classification [30]. We found no cases of type I hydrocele in this study group. Type II hydrocele was found in the majority of cases (94.6%), while type III was found in the remaining 5.4% of cases, which is consistent with some other studies. Ahmed Elhaddad et al. conducted a prospective study of 93 male children with 106 hydroceles in which they found type II hydroceles in 73.6% of cases. Type I was found in only 8.5% and type III in the remaining 17.9% [5]. Our study is consistent with the findings of Choi et al. who described an open internal inguinal ring in all cases [39]. Saka et al. also reported that 97.7% of hydroceles around the internal inguinal ring were patent (59.1% type II and 38.6% type III), with type I found in only one patient (2.2%) [40].

When it comes to the success of hydrocele surgery, one of the most important factors is the number of recurrences. The recurrence rate after laparoscopic hydrocelectomy has been reported in recent studies to be 0–1.4% [9,33]. Reducing the recurrence rate remains a major clinical challenge. In our study, there was no recurrence of hydrocele in any of the study groups. In the study by Elhaddad et al., there was also no evidence of recurrent hydrocele or testicular atrophy in any of the cases [5]. However, the study by Liu et al. showed a significantly higher recurrence rate in the OR group than in the LR group (6.67% vs. 0%, *p* = 0.034) [13]. In the study by Zhang et al., 9 recurrences (1.1%) occurred in the 950 laparoscopic procedures, which did not differ significantly from the 5 cases in the open repair group (1.3%) [8]. In a study by Choi et al., there was also one case of recurrence (0.7%) in the scrotal incisional hydrocelectomy (SIH) group compared with the total laparoscopic hydrocelectomy group in which no cases of recurrence were reported [39].

As with any surgical technique, neither PIRS is not without complications. Our previous study has clearly shown that the pediatric surgeon’s experience with laparoscopic procedures reduces the complication rate to a minimum after 25–30 completed PIRS procedures per surgeon [37]. The most common intraoperative complication is injury to the epigastric or iliac blood vessels [21,36,38]. Special care must be taken when inserting the needle in the abdominal cavity, especially when manipulating the needle around the internal inguinal ring to avoid injury to the surrounding blood vessels [32,41]. In the event of accidental puncture of iliac or epigastric blood vessels, laparoscopic surgery can only be completed if no further bleeding occurs and no retroperitoneal hematoma forms. If this is not possible, open surgery should be performed. In this study, two cases of accidental injury to the epigastric vein were noted in both groups. In all cases, the hematoma was self-limiting and the bleeding stopped spontaneously after short external compression. In the PIRS group, all cases were completed by laparoscopy. Severe pain and swelling of the scrotum were rarely reported. In our study, there were no statistically significant differences in terms of postoperative complications, although the number of postoperative complications was higher in the OR group (*n* = 7, 6.3%) than in the PIRS group (*n* = 2, 2.3%), with the most common complication being scrotal hematoma in OR group (*n* = 4). All postoperative complications were treated conservatively. However, the study by Liu et al. showed a significantly higher persistence of scrotal swelling in the OR group than in the LR group (10% vs. 1.54%, *p* = 0.034) [13]. Zhang et al. also reported a significantly higher incidence of scrotal edema in the OR group than in the LR group (71 (18.6%) vs. 0, *p* < 0.01). In addition, a significantly higher recurrence of incisional infection was found in the OR group (*n* = 6 (1.6%) vs. *n* = 1 (0.1%), *p* < 0.01) [8].

The median operative times reported in the literature for unilateral and bilateral repairs using the PIRS technique in the treatment of pediatric inguinal hernias are 11 to 19 min and 18 to 24 min, respectively [22,36,38]. In this study, the PIRS technique proved to be superior in terms of the duration of the procedure and anesthesia. The median duration of the procedure (15 min vs. 21 min; *p* < 0.001) and anesthesia (30 min vs. 40 min; *p* < 0.001) were significantly lower in the PIRS group compared to open ligation of the PPV. Although this difference was statistically significant, the slightly shorter duration of surgery or anesthesia in the PIRS group is without clinical relevance. In addition, a significantly shorter median length of stay was observed in the PIRS group compared to the open PPV group (9 h vs. 24 h *p* < 0.001). Our results are consistent with the study by Zhang et al. They also showed a shorter operation time in the LR group than in the OP group (16.11 ± 12.21 min vs. 28.42 ± 8.95 min, *p* < 0.01). In addition, a significantly shorter LOS was found in their study (1.08 ± 0.31 vs. 2.73 ± 1.50 days, *p* < 0.01) [8].

In a majority of cases, the choice of surgical technique still depends on the surgeon. This study shows that a minimally invasive approach in the hands of an experienced pediatric surgeon can be a safe and effective technique to use in the treatment of communicating hydrocele in children, as it has shown excellent results, including a low incidence of complications and recurrence. This approach should be offered to parents when deciding on the type of surgery.

Our study has some limitations. The first limitation is a retrospective study design, which means that some information about the patients that could be important for the results is not available. Also, the fact that it is a single-center study means that the results may not be generalizable to the general population. In addition, the follow-up period is relatively short and the sample is not large enough. Finally, there is no randomization, so the study is susceptible to selection bias. The children in the PIRS group were one year younger and had a lower weight and height. We believe that these differences were random and had no influence on the study results. The results of this study would need to be correlated with further analyses based on more pediatric PIRS cases. Our results may provide a basis for further evaluation of the PIRS technique in the treatment of hydrocele in the pediatric population. Further randomized, prospective studies in large samples should be conducted to obtain reliable data.

## 5. Conclusions

To summarize, both techniques are safe and effective in the treatment of communicating hydrocele in children. PIRS showed excellent outcomes and a low incidence of complications and recurrences, comparable to traditional open surgery. However, further studies need to be conducted on this topic.

## Figures and Tables

**Table 1:** Demographic and clinical data of the patients operated on because of hydrocele (*n* = 198).

Variables Median (IQR) or *n* (%)	Group I (*n* = 87)	Group II (*n* = 111)	*p*
	PIRS	Open Surgery	
Age (months)	3 (3, 4)	4 (3, 5)	0.002 *
Weight (kg)	19 (16, 21)	21 (20, 26)	<0.001 *
Height (cm)	111 (101, 117)	120 (111, 131)	<0.001 *
BMI (kg/m^2^)	15.2 (14, 16.4)	15.5 (14.3, 16.3)	0.447 *
Lateralization			
Right	59 (67.8)	70 (63.1)	0.174 ^†^
Left	23 (26.4)	39 (35.1)	
Bilateral	5 (5.8)	2 (1.8)	
ASA classification			0.891 ^‡^
ASA I	82 (94.2)	104 (93.7)	
ASA II	5 (5.8)	7 (6.3)	
Comorbidities	7 (8.1)	7 (6.3)	0.771 ^‡^

* Mann–Whitney U-test; ^†^ Fisher’s exact test; ^‡^ chi-square test; IQR—interquartile range; PIRS—Percutaneous Internal Ring Suturing; BMI—Body Mass Index; ASA—American Society of Anesthesiologists.

**Table 2:** Classification of hydrocele by laparoscopy in the PIRS group according to Chang’s classification [30].

Type of Hydrocele	Group I (*n* = 92) *
	PIRS; *n* (%)
Type I (Closed ring—No communication to PC)	0 (0)
Type II (Patent ring—Direct communication to PC)	
Type II A (Wide opening)	69 (75)
Type II B (Ring covered by peritoneal seal)	4 (4.3)
Type II C (Pinhole)	14 (15.3)
Type III (Patent ring—No communication to PC)	
Type III A (Solitary cyst)	4 (4.3)
Type III B (Multiple cysts)	1 (1.1)

* In five cases bilateral repair was performed (*n* = 92): PC—Peritoenal Cavity; PIRS—Percutaneous Internal Ring Suturing.

**Table 3:** Outcomes of treatment of the patients operated on for hydrocele (*n* = 198).

Variables Median (IQR) or *n* (%)	Group I (*n* = 87) ^‡^	Group II (*n* = 111) ^‡^	*p*
	PIRS	Open Surgery	
Duration of surgery (min)	15 (10, 17)	21 (15, 25)	<0.001 *
Duration of anesthesia (min)	30 (25, 40)	40 (35, 40)	<0.001 *
Intraoperative complications			>0.999 ^†^
Epigastric vein injury	2 (2.3)	2 (1.8)	
Postoperative complications	2 (2.3)	7 (6.3)	0.190 ^†^
Swelling in groin	1	0	
Scrotal edema	1	2	
Scrotal hematoma	0	4	
Wound infection	0	1	
Length of hospital stay (h)	9 (8, 12)	24 (12, 24)	<0.001 *
Recurrence	0 (0)	0 (0)	-
Follow-up (months)	30 (15, 38)	32 (15, 49)	0.395 *

* Mann–Whitney U-test; ^†^ Fisher’s exact test; ^‡^ in five cases in PIRS group (*n* = 92) and in two cases in open group (*n* = 113) bilateral repair was performed.

## Data Availability

The data assessed and reported here can be obtained from the authors upon reasonable request and following ethical and privacy principles.
